# Phytochemical Analysis and Genotoxicological Evaluation of Prickly Pear Peel Extracts

**DOI:** 10.3390/plants12071537

**Published:** 2023-04-03

**Authors:** Margarita Dormousoglou, Ioanna Efthimiou, Maria Antonopoulou, Stefanos Dailianis, Giulia Herbst, Dimitris Vlastos

**Affiliations:** 1 Department of Sustainable Agriculture (Former Department of Environmental Engineering), University of Patras, Seferi 2, GR-30100 Agrinio, Greece; 2 Department of Biology, University of Patras, GR-26500 Patras, Greece; 3 Department of Chemical Engineering, Federal University of Paraná, Curitiba 81531-990, PR, Brazil

**Keywords:** prickly pear peel, *Opuntia ficus-indica*, plant extracts, CBMN assay, genotoxicity, antigenotoxicity, cytotoxicity, antioxidant activity, mitomycin C, human lymphocytes

## Abstract

This study investigated the beneficial properties of prickly pear peel (PPP) extracts from *Opuntia ficus-indica* (L.) Mill. Extracts were obtained via the Soxhlet extraction method using methanol (P1), ethanol (P2) and ethanol-water (P3) as extraction solvents. Their total phenolic and flavonoid content (TPC and TFC, respectively) and their antioxidant activity (AA) were determined. The PPP extracts were characterized in detail using mass spectrometry techniques. Their cyto-genotoxic effect and antigenotoxic potential against mitomycin C were evaluated via the cytokinesis block micronucleus (CBMN) assay on human lymphocytes. Enhanced TPC, TFC and AA values were recorded for all the extracts. Moreover, P1 and P2 were cytotoxic only at the highest concentrations, whereas P3 was found to be cytotoxic in all cases. No significant micronucleus induction was observed in the tested extracts. The PPP extracts contain bioactive compounds such as flavonoids, carboxylic acids, alkaloids, fatty acids and minerals (mainly K, Si, Mg, Ca, P and Zn). The results showed that all three extracts exerted high antigenotoxic activity. Our findings confirm the beneficial and genoprotective properties of PPP extracts and further studies on the bioactive compounds of *Opuntia ficus-indica* (L.) Mill. are recommended, as it constitutes a promising plant in pharmaceutical applications.

## 1. Introduction

Plants have been characterized as an important source of pharmaceutical products with therapeutic or prophylactic properties which are associated with anti-inflammatory, antioxidant, antibacterial, antigenotoxic and antiproliferative activities of their bioactive compounds [1,2,3,4]. Due to their beneficial properties, their use for various therapeutic purposes has increased tremendously over the last decades worldwide. Presently, many plants are used as a source of ingredients to prevent and/or treat a plethora of diseases [5]. Among them, *Opuntia ficus-indica* (L.) Mill., also known as prickly pear or cactus, belonging to the Cactaceae family and *Opuntia* genus, is of great interest due to its bioactive compounds and their potential valuable attributes [6,7].

*Opuntia ficus-indica* grows mainly in Latin America and Mexico, as well as South Africa and Mediterranean countries such as Spain and Greece [7,8,9,10]. The plant consists of cladodes, flowers, fruit and seeds and contains many biologically active substances including minerals, phenolic acids and flavonoids, [7,10,11,12]. In recent years there has been great interest in the fruit (prickly pear) of *Opuntia ficus-indica* due to its increased consumption fresh or as a processed product (e.g., juices and jams), which gives it a higher commercial value. Additionally, prickly pear is widely used in cosmetics, biofuel production and animal nutrition as well as for medical purposes [13,14,15,16]. Prickly pear fruit peel comprises a high percentage of the fruit ~45% to 50% and consequently an annual high quantity of waste generated and discarded due to its consumption and processing is inevitable. However, this kind of waste constitutes a significant source of high-added value products with many potential applications [11]. Thus, *Opuntia ficus-indica* waste seeds, resulting from the extraction of oil have been used for the manufacturing of active carbon for dye removal [17], as a sustainable lignocellulosic source [18] and to partially replace phenol formaldehyde resins to produce plywood composites [19]. Moreover, *Opuntia ficus-indica* fruit peels are deemed an exceptional carbon source for the production of lactic acid [20].

It is well known that peels and pomace of fruits constitute primary waste of the agri-food industry with high content of phenolic compounds [21]. According to Lizárraga-Velázquez et al. [22], plant wastes such as prickly pear peel represent a significant and low-cost source of antioxidants (terpenes, phenolic compounds, phytosterols) with potential applications as pharmaceutical products due to the antidiabetic, antihypertensive, anticancer and antibacterial properties they may possess [23,24]. Cladode extract of *Opuntia ficus-indica* induced antimicrobial activity against Gram-negative and Gram-positive bacteria, in addition to exerting antibiofilm activity against *Staphylococcus aureus* [25]. Prickly pear seed oil had a similar action against bacteria while displaying antifungal activity against *Saccharomyces cerevisiae* and *Candida albicans* [26]. In addition, prickly pear peel extract was found to possess significant potential against pneumonia pathogens [11]. Chronic inflammation induced in mice was mediated by the anti-inflammatory activity exhibited by the methanolic extract of prickly pear stem and was ascribed mainly to β-sitosterol [27]. Moreover, inflammatory response caused by alcohol consumption was attenuated by prickly pear juice [28]. The hepatoprotective effect of *Opuntia ficus-indica* cladode extract against lithium poisoning in rats was demonstrated by a significant increase of the hepatic catalase, superoxide dismutase and glutathione peroxidase activities [29]. Similarly, polysaccharides from prickly pears showed a liver protective action against organophosphorus pesticides [30]. *Opuntia ficus-indica* f. *inermis* juice had a hepatoprotective effect which was attributed to increased antioxidant activity [31]. The cardioprotective potential of *Opuntia ficus-indica* powdered cladodes was demonstrated against the development of atherosclerotic lesions in apoE-KO mice by Garoby-Salom et al. [32]. A rapid increase of HDL cholesterol levels and simultaneous decrease of LDL and triglycerides in women with metabolic syndrome was reported after consumption of prickly pear dried leaves [33]. Different parts of prickly pear have been tested for their chemopreventive and antigenotoxic potential. Namely, Helal et al. [34] recorded a concentration-dependent inhibition of the proliferation of lung (A549) and colon (Caco2) cancer cells following treatment with prickly pear extract. A similar pattern was observed against liver cancer cells (HepG2), colorectal adenocarcinoma (Caco-2) and breast cancer cell line (MCF-7), after treatment with increasing concentrations of prickly pear extract [35]. Furthermore, methanol extract of *Opuntia ficus-indica* peel exerted antiproliferative activity against breast (MDA-MB-231) and liver (HepG2) cancer cell lines [36]. According to the results of several studies, the antigenotoxic activity of prickly pear parts is attributed to their antioxidant capacity, the inhibition of cell proliferation and induction of apoptosis, their pro-oxidant activity and lipid peroxidation modulation, among others ([37] and references therein).

Considering that prickly pear peel extracts could constitute a considerable natural product for medicinal applications, the present study focused on an investigation of the potential beneficial effects of *Opuntia ficus-indica* by determining the antioxidant, cytotoxic and antigenotoxic effects of three different extracts from the peel of prickly pear fruit. All the extracts were also fully characterized using a combination of mass spectrometry techniques. To the best of the authors’ knowledge, this is the first study assessing the cyto-genotoxic potential of prickly pear peel extracts against mitomycin C (MMC) in cultured human lymphocytes, via a CBMN assay.

## 2. Results

### 2.1. Extraction Yields

The overall Soxhlet yields were obtained over a 6 h period with different solvents (Table 1). The highest extraction yields were for the P1 and P3 extracts with percentages of 66.1 ± 1.4 and 61.2 ± 3.8 % wt. The P2 extract had the lowest yield with a value of 35.3 ± 0.8 % wt.

### 2.2. TPC, TFC and AA

#### 2.2.1. TPC and TFC

The P3 extract showed the highest TPC value (27.46 ± 2.35 mg GAE g^−1^), followed by the P1 extract (22.68 ± 2.21 mg GAE g^−1^) (Table 2), whereas the P3 extract had the lowest TPC value (15.70 ± 0.80 mg GAE g^−1^). As far as TFC is concerned, the highest value was recorded for the P2 extract (2.73 ± 0.06 mg CE g^−1^), followed by the P3 (1.77 ± 0.17 mg CE g^−1^) and P1 (1.70 ± 0.07 mg CE g^−1^) extracts.

#### 2.2.2. Antioxidant Activity (AA) 

To identify the AA of all extracts, ABTS, DPPH and FRAP assays were used (Table 3). The P3 extract had the most pronounced AA in the ABTS assay. The P2 and P3 extracts demonstrated higher AA in DPPH and FRAP assays compared to P1, with the P2 extract showing the highest values.

### 2.3. Characterization of Prickly Pear Peel Extracts by ICP-MS/MS, GC-MS and UHPLC-MS

In the present study, various minerals/elements (Mg, Al, Si, K, Ca, P, Mn, Fe, Cu and Zn) of prickly pear peel were determined and then quantified for all three extracts by ICP-MS/MS analysis. Table 4 shows the results of Mg, Al, Si, K, Ca, P, Mn, Fe, Cu and Zn contents determined by ICP-MS/MS in all extracts. In all cases K and Si had the highest concentrations, with values ranging from 11,423.07 to 25,071.58 μg g^−1^ (P1–P3) and 1337.33 to 1452.05 μg g^−1^ (P1–P3), respectively. In addition, high levels of Mg, Ca, P and Zn, but lower levels of Al, Mn, Fe and Cu were detected in all extracts.

Both GC-MS and UHPLC-MS analyses of the extracts (P1, P2 and P3) were performed and 11 compounds (Table 5) were identified in all cases.

In the case of the GC-MS analysis, the compounds that were identified were (a) trans-cinnamic acid, (b) debrisoquine and (c) n-hexadecanoic acid (palmitic acid). The acquired spectra (Figure S1) were compared with the existing ones in the NIST library. With regard to the UHPLC-MS analysis, eight compounds were detected (tri-glycosylated kaempferol; tri-glycosylated methyl-quercetin derivative I; tri-glycosylated methyl-quercetin derivative II; tri-glycosylated quercetin I; tri-glycosylated quercetin II; di-glycosylated quercetin (rutin); di-glycosylated methyl-quercetin I; di-glycosylated methyl-quercetin I) and their structural assignment was conducted in accordance with the formation of molecular mass ions ([M-H]^−^) and their fragments (where available) following comparison to data already reported in past research [40]. Figure S2 depicts the mass spectra of the identified compounds identified by UHPLC-MS. According to the results, flavonoids, carboxylic acids, alkaloids, and fatty acids were mainly present.

### 2.4. CBMN Assay in Human Lymphocytes

**Figure 1:**
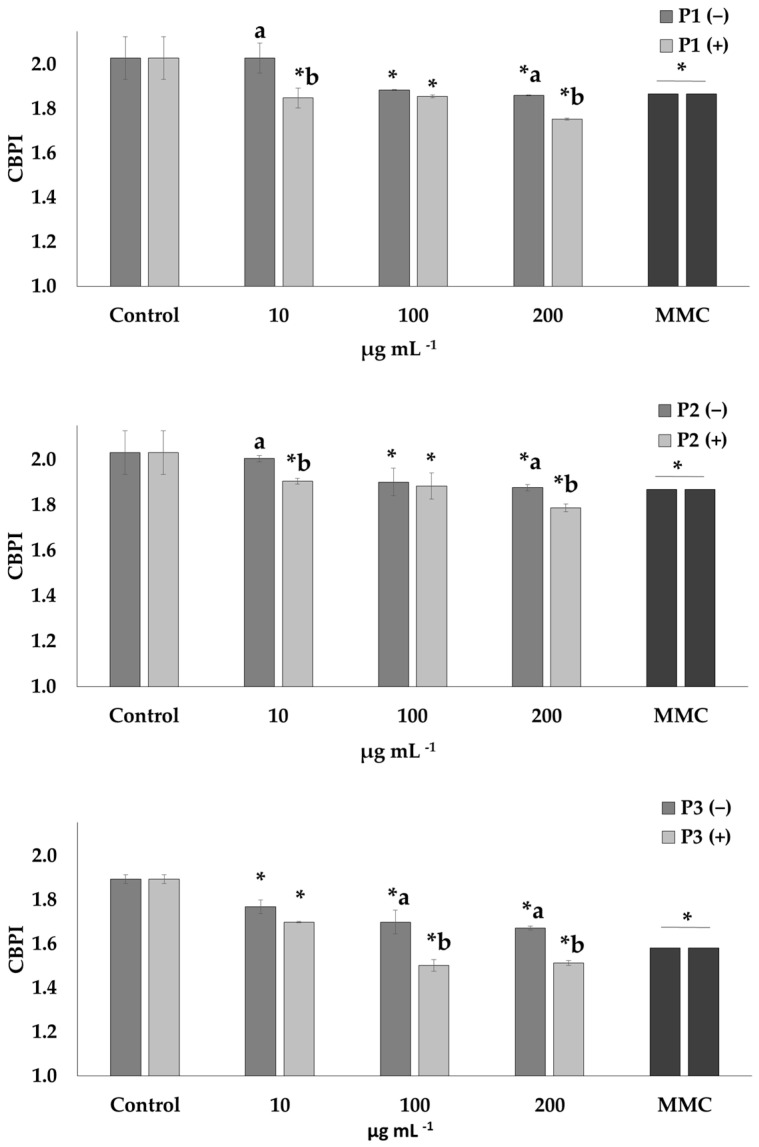
Cytotoxic activity (CBPI values) of P1, P2 and P3 extracts of prickly pear peel in human lymphocytes in the presence (+) and absence (−) of mitomycin C (MMC, 0.5 μg mL^−1^). Values with asterisk (*) significantly differ from the control. The values designated by the different letters are significantly different (Mann–Whitney U-test, *p* < 0.05).

**Figure 2:**
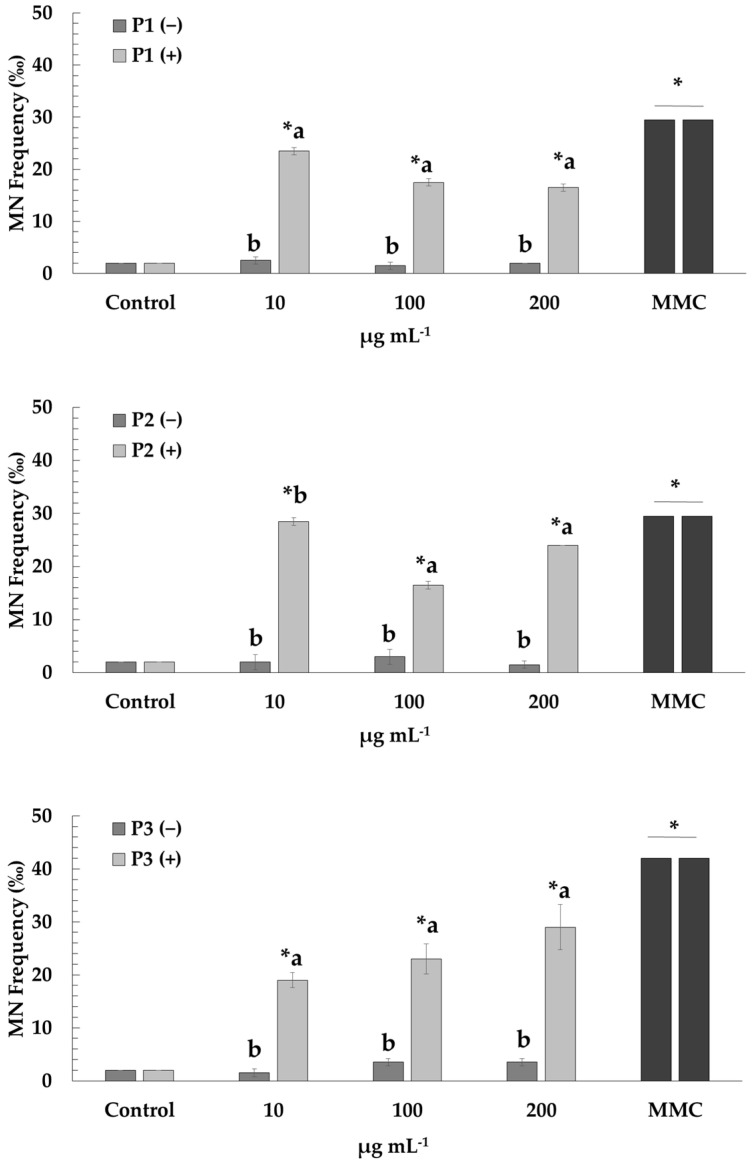
Micronuclei induction in human lymphocytes after treatment with P1, P2 and P3 extracts of prickly pear peel in the presence (+) and absence (−) of mitomycin C (MMC, 0.5 μg mL^−1^). Values with asterisk (*) significantly differ from the control. The values designated by the different letters are significantly different (Mann–Whitney U-test, *p* < 0.05).

The cyto-genotoxic potential of all prickly pear peel extracts was assessed at three concentrations (10, 100 and 200 μg mL^−1^). Afterwards, their capability to diminish MMC-mediated detrimental effects on human lymphocytes was investigated (Figure 1 and Figure 2). The cytotoxicity of the extracts in the presence and absence of MMC was determined through CBPI (Figure 1). 

The P1 and P2 extracts were cytotoxic at the highest concentrations (100 and 200 μg mL^−1^), while for P3, higher rates of cytotoxicity were observed compared to the other two extracts at all tested concentrations (10–200 μg mL^−1^). Cells treated with MMC, in the presence and absence of the extracts, exhibited cytotoxic potential in all the concentrations (10–200 μg mL^−1^).

Regarding the genotoxic activity of the extracts (P1, P2 and P3), low frequencies of MN formation were observed at all tested concentrations (10–200 μg mL^−1^), compared to those that occurred in the control culture, thus indicating lack of genotoxicity. However, cells treated with MMC showed increased MN frequencies as expected, confirming its genotoxic action. Mixtures of MMC with P1 and P3 extracts led to the diminution of MMC-mediated genotoxic effects at all concentrations (10–200 μg mL^−1^). A similar pattern was reported for mixtures of MMC with P2 extract at the two highest concentrations (100 and 200 μg mL^−1^) (Figure 2).

## 3. Discussion

### 3.1. TPC, TFC and AA of Extracts

In the present study, extracts from prickly pear peel using three different solvents (methanol, ethanol, and ethanol-water; ratio 4:1) were prepared using the Soxhlet method. The evaluation of the yields of the extracts in relation to the used solvents, revealed that methanol and ethanol-water led to the highest yields compared to ethanol, and the results are in accordance with the yields observed for the same solvents in previous studies [41,42]. Other factors that affect the extraction yield and can be considered are geographical origin, harvest season and fruit ripeness [43,44,45].

All the studied extracts exhibited high TPC and TFC values. Regarding TPC content the following order P3 > P1 > P2 was observed. The observed values are in accordance with the reported values for the respective solvents as stated by Abou-Elella and Ali [42]. These findings indicate that phenolic compounds are often extracted in higher amounts in more polar solvents such as a mixture of water-ethanol compared with methanol or ethanol [46,47,48]. Our results regarding TFC contradict those observed by Abou-Elella and Ali [42] as a higher flavonoid content was found when EtOH was used as solvent (P2 > P3 > P1, EtOH > EtOH-W > MeOH, respectively).

In our efforts to evaluate the antioxidant activity (AA) of all the obtained extracts in this study we implemented the ABTS, DPPH and FRAP assays. The obtained results reveal that the highest AA values were observed in P2 and P3 extracts, followed by P1 extract, following the same pattern as the findings of Abou-Elella and Ali, [42]. In the case of ABTS, the highest antioxidant activity was induced by the P3 extract. However, P2 had the highest AA at the DPPH and FRAP assays. Τhis could be attributed to certain variations among the methods used such as the different absorbance of the radicals. The maximum absorbance of the ABTS radical is at 734 nm in an ethanolic medium, DPPH has an absorbance peak at 517 nm in a methanol solution and FRAP at 593 nm in a sodium acetate buffer solution [49,50,51]. The free radical scavenging activities of the extracts depend on the capacity of the antioxidant compounds that they contain, such as flavonoids and polyphenols [52]. Various studies in the literature report high TPC and TFC values as well as significant antioxidant activity of the different extracts of prickly pear [7,53,54,55,56,57].

The observed differences between TPC, TFC and AA values can be attributed to various factors related to the geographical origin of the plant as well as the microclimate and environmental conditions of the area. In addition, the experimental procedures and conditions applied during the extraction process in order to produce the extracts may affect the distribution of the TPC, TFC and AA values [41,58,59,60].

### 3.2. Cyto-Genotoxic Potential of the Extracts

The cytotoxic and genotoxic potential of prickly pear peel extracts (P1, P2 and P3) was evaluated via the CBMN assay.

The observed results on the potential cytotoxic activity of the studied extracts showed a slight decrease in CBPI values at the highest concentrations (100 and 200 μg mL^−1^) of P1 and P2 extracts, a fact that demonstrates a mild cytotoxic effect. In contrast, a high decrease in CBPI values was observed at all tested concentrations (10–200 μg mL^−1^) of P3. Despite the absence of data on the cytotoxicity of prickly pear peel, the high cytotoxicity could be explained by the individual compounds of extracts such as total phenolics, tannins and betalains (i.e., betacyanins and betaxanthins). According to Abou-Elella and Ali [42], the ethanol, methanol and ethanol-water (80:20) extracts of prickly pear peel are rich in polyphenols, tannins and betalains (betacyanins and betaxanthins) and the highest amounts of tannins and betalains were found in the ethanol-water extract. Studies on different cell lines such as a human chronic myeloid leukemia cell line (K562) and HepG2 cells demonstrated that betalains can induce apoptotic and cytotoxic effects, respectively [61,62]. Furthermore, tannins demonstrated cytotoxic effects in human lymphocytes [63] and extracts with high quantities of polyphenols have been reported to induce cytotoxic activity on human and mouse tumor cell lines [64,65].

Our data clearly indicate that treatment with different concentrations of prickly pear peel extracts (P1, P2 and P3) does not induce genotoxic effects (in terms of MN formation) in cultured human lymphocytes. Similar results in in vivo experiments in mice and rats, regarding the absence of genotoxicity, have been reported for extracts from other parts (cladodes and stems) of the *Opuntia ficus-indica* plant which is in line with our findings [6,66,67]. In addition, Madrigal-Santillán et al. [68] reported that treatment with prickly pear juice did not show any genotoxic potential in mice (strain NIH). 

### 3.3. Cytoprotective and Antigenotoxic Effects of Prickly Pear Peel Extracts against Mitomycin C (MMC)

Considering that the compounds contained in plant extracts may reduce the damage caused by mutagens, the possible antigenotoxic/antimutagenic effects of prickly pear peel extracts against mitomycin C (MMC) were examined in human lymphocytes.

When applying a CBMN assay, MMC is recommended as a positive control by OECD protocol [69]. Mitomycin C can influence DNA synthesis by binding complementary helices found within it [70,71]. Furthermore, free radicals formation and the ability of MMC to alkylate guanine residues, induce MN formation [72,73], the presence of which is closely linked to tumor progression and carcinogenesis [74].

A significant increase in MN formation was induced by MMC as compared to the control, and the observed results are in accordance with previous studies [41,70,75,76,77]. A significant decrease of the genotoxic impact of MMC is reported for the first time, in the presence of P1 (concentrations, 10–200 μg mL^−1^), P2 (concentrations, 100 and 200 μg mL^−1^) and P3 (concentrations, 10–200 μg mL^−1^) prickly pear peel extracts, with the highest antigenotoxic rates observed in P1 and P3 extracts. The effectiveness of the specific extracts against MMC-mediated genotoxicity could be due to the presence and action of various beneficial compounds, such as flavonoids, phenolic acid and minerals, among others. In addition, the higher TPC values exhibited by the P1 and P3 extracts could partially explain their higher antigenotoxic activity as the beneficial properties of the extracts are linked to the presence of secondary metabolites. Despite the absence of data on the antigenotoxicity of prickly pear peel, there are few studies on the potential antigenotoxic activities of extracts from different parts of *Opuntia ficus-indica* against well-known genotoxic and/or mutagenic agents. Siriwardhana et al. [78] applying the comet assay, showed that a prickly pear fruit extract reduced the H_2_O_2_-induced DNA damage in human peripheral lymphocytes and the observed reduction could be associated with the extract constituents (mainly flavonoids and betalains). In vivo experiments in mice clearly demonstrated the protective and antigenotoxic activity of *Opuntia ficus-indica* cladode extracts against Aflatoxin B1 and the mycotoxin Zearalenone, probably due to their ability to promote the antioxidant defense system and inhibit the oxidative process induced by the aforementioned genotoxic/mutagenic agents [66,67].

This pattern could be associated with the chemical composition of the extracts and mainly the identified phenolic compounds and flavonoids. The main components were found to be n-hexadecanoic acid (palmitic acid), trans-cinnamic acid, debrisoquine, tri-glycosylated kaempferol, tri-glycosylated methyl-quercetin derivative I, tri-glycosylated methyl-quercetin derivative II, tri-glycosylated quercetin I, tri-glycosylated quercetin II, di-glycosylated quercetin (rutin), di-glycosylated methyl-quercetin I, and di-glycosylated methyl-quercetin II. Phenolic compounds and flavonoids have been reported to possess antioxidant, anti-inflammatory, anticancer and hepatoprotective activities [79].

Barcelos et al. [80] demonstrated that flavonoid quercetin and its derivative rutin cannot induce genotoxic effects in the examined concentrations. On the other hand, both flavonoids reduced DNA damage induced by genotoxic/mutagenic agents in HepG2 cells using the comet assay. The previous study confirmed the findings by Ramos et al. [81] about the antiproliferative activity of quercetin in the same cell line against tert-butyl hydroperoxide (t-BHP). Kaempferol was able to modulate and decrease the cytotoxic and genotoxic/clastogenic effect of zeocin in human lymphocytes [82] and to exert inhibitory activity against genotoxic/mutagenic agents via the SOS chromotest bacterial assay system in the presence of *Escherichia coli* PQ37 strain [83]. Prickly pear peel extracts also contain palmitic acid known for its antimutagenic/anticlastogenic properties [84].

Micronutrients are critical for DNA stability as well as in cellular processes. Certain micronutrients such as the minerals which were detected in the studied extracts (i.e., Mg, Si, Al, K, Ca, P, Mn, Fe, Cu and Zn) have been reported to play a key role in cellular processes. Moreover, the interactions and synergistic effects among the various components that coexist in natural products play a fundamental role to their potential positive impact ([41,70,77,85,86] and references therein).

## 4. Materials and Methods

Prickly pear peel extracts were primarily prepared using different extraction solvents (methanol, ethanol, and ethanol-water ratio 4:1) by the Soxhlet method, widely used for the extraction of bioactive compounds from natural products, as it leads to high extraction yields and bioactivity of the extracted molecules [87,88]. The DPPH, FRAP and ABTS tests were used to determine the total phenolic and flavonoid content (TPC and TFC) and antioxidant activity (AA) of the obtained extracts. Afterwards, the cyto-genotoxic potential of each extract, in addition to the cyto/genoprotective activity against mitomycin C (MMC) were investigated in cultured human lymphocytes, via the cytokinesis block micronucleus (CBMN) assay, which is a broadly applied and reliable method for the assessment of the cyto-genotoxic profile of chemicals [41,75,76,77,89]. The MMC is isolated from *Streptomyces caespitosus* and has been used as a chemotherapeutic agent as well as a genotoxic inducer in a plethora of antigenotoxicity studies [41,69,75,76,77,85].

### 4.1. Chemicals and Reagents

Ham’s F-10 medium, foetal bovine serum (FBS), phytohemagglutinin (PHA) and L-glutamine were purchased from Gibco, (Fisher Scientific, Loughborough, Leicestershire, UK Ltd.). Mitomycin C (MMC) and Giemsa were commercially procured from Sigma-Aldrich Chemical Co. (St. Louis, MO, USA) and cytochalasin-B (Cyt-B) from Santa Cruz Biotechnology (Heidelberg, Germany). The remaining solvents and chemicals used were of the highest grade commercially available. All stocks of the compounds and solutions were stored at 4 °C until use. The primary stocks of the tested compounds were utilized for the treatments. 

### 4.2. Sample Preparation

The harvest of the *Opuntia ficus-indica* fruit took place at the Aitoloakarnania region, Western Greece (coordinates: 38°24′2″ N 21°50′8″ E) in September 2020, where the ambient temperature was between 30 to 35 °C and the fruit was at physiological maturity. After harvesting, the fresh fruits were transferred to the laboratory and plant identification carried out. The Department of Biology Herbarium at the University of Patras possesses the voucher specimen of *Opuntia ficus-indica* (39988 UPA-Herbarium) which was deposited there for reference purposes. 

The fruits were washed under tap water with a brush for 2 min and the peels from the fruits were removed manually [7,90]. The peels were cut into small pieces and dried in a forced air oven at 60 °C for 24 h [91,92]. After drying, peel was ground in a domestic food processor for 10 s to determine the average particle size. This was accomplished in a vertical vibrating sieve shaker using Tyler series sieves with apertures of 8, 10, 20, 28, 32 and 35 mesh [93]. The average particle size (PS) obtained was 0.57 ± 0.02 mm. The raw material was packed in plastic bags and stored at −20 °C until use.

#### Soxhlet Extraction

Soxhlet extraction was applied according to the method 920.39 of AOAC [94] and the procedure followed has been previously described [41] (see Section S4.2)

### 4.3. Determination of TPC, TFC, and AA

#### 4.3.1. Preparation of Samples

A volume of 0.8 mL of methanol:H_2_O (80:20 *v*/*v*) was added to 0.2 mL of each extract (P1–P3) and the solutions were vigorously agitated for 5 min before centrifugation (3000 rpm, 10 min). The supernatant was used to determine the total phenolic content (TPC), total flavonoid content (TFC) and the antioxidant activity (AA). All samples were prepared and analyzed in triplicate.

#### 4.3.2. Total Phenolic and Flavonoid Content (TPC and TFC) and Antioxidant Capacity (AA) Determination

The experimental process for the determination of total phenolic and flavonoid content in all extracts (P1–P3) was performed according to Dormousoglou et al. [41] (see Sections S4.3.1 and S4.3.2). Accordingly, the antioxidant activity (AA) was performed through three methods, using the extract solubilized in a methanol-water solution (80% *v*/*v*) as previously described [41] (see Section S4.3.3).

### 4.4. Characterization of Prickly Pear Peel Extracts by ICP-MS/MS, GC-MS and UHPLC-MS

A thorough characterization of all the extracts (P1–P3) was conducted using mass spectrometry techniques, i.e., ICP-MS/MS, GC-MS and UHPLC-MS.

#### 4.4.1. ICP-MS/MS Analysis

An Agilent 8900 Triple Quadrupole ICP-MS/MS (Agilent Technologies, Tokyo, Japan) was used for the detection of the minerals/elements of P1, P2 and P3 extracts according to the testing protocols of ISO 17294 [95,96]. Their quantification was based on calibration curves (R^2^ > 0.999) prepared by an analysis of calibration standards. Before analysis, preparation of the samples took place in an acid matrix (2.5% *v*/*v* HNO_3_ and 0.5% *v*/*v* HCl). Minerals concentration was calculated and expressed as dry weight in μg g^−1^ [97].

#### 4.4.2. GC-MS Analysis

A volume of 2 μL of each extract was injected in splitless mode and analyzed by GC-MS (Agilent 5975B MS coupled to an Agilent 6890N GC) using an Agilent 19091S-433 HP-5MS (5-% phenyl methyl siloxane) of 30 m length, 250 μm diameter and 0.25 μm film thickness analytical column. The appropriate parameters were implemented (see Section S4.4.1).

#### 4.4.3. UHPLC/MS Analysis

An Ultimate 3000 RSLC System (Thermo Fisher Scientific, Waltham, MA, USA) coupled to an amaZon SL ion trap mass spectrometer (Bruker, Bremen, Germany) with an ESI source was employed for further analysis of the extracts, with an injection volume of 5 μL. Appropriate parameters were applied (see Section S4.4.2).

### 4.5. CBMN Assay in Human Lymphocytes In Vitro

#### 4.5.1. Ethics Statement

The specific research received approval from the Research Ethics Committee (REC) of the University of Patras (UPAT) (Ref. No. 11584/6 March 2018). Blood samples were acquired from two healthy non-smoking male donors (20 and 25 years old), who declared that they had not been recently exposed to radiation, drug treatment or any viral infection. Thereafter, samples were handled and treated appropriately for conducting the CBMN assay.

#### 4.5.2. CBMN Assay Application

The cytotoxic, genotoxic and antigenotoxic potential of prickly pear peel extracts (P1–P3) in human lymphocytes was determined by the in vitro cytokinesis block micronucleus (CBMN) assay using cytochalasin-B (see Section S4.5.1, Figure S3), according to standard procedures [69].

Cytotoxicity was determined using the cytokinesis block proliferation index (CBPI), by counting 1000 cells for each experimental point and using the equation below:

CBPI = [N1 + N2 + 3(N3 + N4)]/N,(1)

where N1, N2, N3 and N4 represent the numbers of cells with one, two, three and four nuclei, while N is the total number of cells [98].

### 4.6. Statistical Analysis

All data are expressed as mean ± standard deviation of three independent experiments in each case. The data sets of the extraction yields TPC, TFC, ABTS, DPPH, FRAP, observed in each extract (P1–P3), as well as the CBPI and MN frequency values observed in challenged cells after exposure to different concentrations of each extract (P1–P3), were checked for homogeneity of variance (Levene’s test of equality of error variances) and assumptions of normality (Shapiro-Wilk W Test) via the SPSS 25 (IBM Inc., Armonk, NY, USA, 2019) software package. Thereafter, a one-way ANOVA was performed to determine statistical variance among groups for each chemical parameter tested, followed by a post hoc analysis (Bonferroni test) to estimate statistically significant differences among extracts in each case. Regarding the CBPI and MN frequency values observed in challenged cells, statistical variance among groups (MMC-treated and MMC-free cells, treated with different concentrations of P1–P3) were assessed non-parametrically via the Kruskal-Wallis test, while statistically significant differences among MMC-treated and MMC-free cells were evaluated by the use of the Mann–Whitney U-test. Significant levels were established as *p* < 0.05 in all cases.

## 5. Conclusions

Τhe results of the present study reveal significant findings on the antioxidant, cytotoxic, genotoxic and antigenotoxic profile, from three extracts of prickly pear peel. According to the results, all extracts (P1, P2 and P3) exhibited satisfactory and relatively high content of phenolics and flavonoids, while the highest AA values were observed in P2 and P3 extracts, followed by P1 extract. The significant antigenotoxic potential demonstrated by all the studied extracts (P1, P2 and P3) was verified for the first time. All the extracts induced cytotoxic effects with P3 inducing the highest cytotoxicity. The chemical composition of the extracts and mostly the presence of phenolic compounds and flavonoids could potentially lead to the observed properties. Simultaneously, the valorization of prickly pear’s peel for the formation of products with beneficial properties is highlighted. Considering the antioxidant potential and the remarkable protective effects of prickly pear peel extracts against the mutagenic agent MMC, the opportunity arises for their further assessment and implementation in pharmaceutical products and medicinal applications.

## Figures and Tables

**Table 1:** Soxhlet extraction yields of prickly pear peel using different solvents. The values designated by the different letters are significantly different (Bonferroni test, *p* < 0.05).

Extraction	Polarity ^#^	Time (Min)	Yield (%) *
P1	6.6	360	66.1 ± 1.4 ^a^
P2	5.2	360	35.3 ± 0.8 ^b^
P3	7.1	360	61.2 ± 3.8 ^a^

^#^ References [38,39]. * Extraction yield expressed in % wt (mean ± standard deviation).

**Table 2:** Total phenolic content (TPC) and total flavonoid content (TFC) of prickly pear peel extracts with different solvents. The values designated by the different letters are significantly different (Bonferroni test, *p* < 0.05).

Extract	TPC (mg GAE g^−1^)	TFC (mg CE g^−1^)
P1	22.68 ± 2.21 ^a^	1.70 ± 0.07 ^b^
P2	15.70 ± 0.80 ^b^	2.73 ± 0.06 ^a^
P3	27.46 ± 2.35 ^a^	1.77 ± 0.17 ^b^

**Table 3:** Antioxidant activities (AA) of prickly pear peel extracts via ABTS, DPPH, and FRAP assays. The values designated by the different letters are significantly different (Bonferroni test, *p* < 0.05).

Extract	AA (μmol TE g^−1^)		
	ABTS	DPPH	FRAP
P1	48.08 ± 7.94 ^ab^	39.16 ± 6.04 ^b^	21.19 ± 3.00 ^b^
P2	27.44 ± 10.87 ^b^	59.53 ± 6.74 ^a^	30.11 ± 4.25 ^a^
P3	54.05 ± 8.81 ^a^	49.43 ± 8.44 ^ab^	27.82 ± 3.89 ^ab^

**Table 4:** Concentrations of minerals/elements in P1, P2 and P3 extracts.

Element	Concentration (μg g^−1^)		
	P1	P2	P3
**Mg**	395.31	154.01	368.98
**Al**	41.27	37.98	42.50
**Si**	1337.33	1399.92	1452.05
**K**	20,604.77	11,423.07	25,071.58
**Ca**	333.27	260.25	402.12
**P**	210.31	141.27	191.36
**Mn**	3.59	2.35	5.89
**Fe**	6.39	19.20	55.44
**Cu**	7.78	10.68	8.86
**Zn**	120.21	185.52	210.75

Mg: magnesium; Al: aluminium; Si: silicon; K: potassium; Ca: calcium; P: phosphorus; Mn: manganese; Fe: Iron; Cu: copper; Zn: zinc.

**Table 5:** Compounds identified by GC-MS and UHPLC-MS in P1, P2 and P3 extracts.

GC/MS	Proposed Compound	MW	P1	P2	P3
	trans-Cinnamic acid	148.2	√	√	√
	Debrisoquine	175.2	√	√	√
	n-Hexadecanoic acid(Palmitic acid)	256.4	√	√	√
**UHPLC/MS**	**Proposed Compound**	**[M-H]^−^**	**P1**	**P2**	**P3**
	Tri-glycosylated kaempferol	739	√	√	√
	Tri-glycosylated methyl-quercetin derivative I	769	√	√	√
	Tri-glycosylated methyl-quercetin derivative II	769	√	√	√
	Tri-glycosylated quercetin I	755	√	√	√
	Tri-glycosylated quercetin II	755	√	√	√
	Di-glycosylated quercetin (Rutin)	609	√	√	√
	Di-glycosylated methyl-quercetin I	623	√	√	√
	Di-glycosylated methyl-quercetin II	623	√	√	√

## Data Availability

Data are contained within the article.

## Supplementary Materials

The following supporting information can be downloaded at: https://www.mdpi.com/article/10.3390/plants12071537/s1, Figure S1: Mass spectra obtained by GC-MS analysis of (a) trans-cinnamic acid, (b) debrisoquine, (c) n-hexadecanoic acid (palmitic acid). Figure S2: Mass spectra obtained by UHPLC-MS in negative ionization mode of (a) tri-glycosylated kaempferol (b) tri-glycosylated methyl-quercetin derivative I (c) tri-glycosylated methyl-quercetin derivative II (d)tri-glycosylated quercetin I (e)tri-glycosylated quercetin II (f) di-glycosylated quercetin (rutin), (g) di-glycosylated methyl-quercetin I, (h) di-glycosylated methyl-quercetin I. Figure S3: Schematic representation of CBMN assay. Refs. [49,50,51,99,100] cited in Supplementary Materials.
