# Supplementary material for: Phytochemical Analysis and Genotoxicological Evaluation of Prickly Pear Peel Extracts
Source: Plants (Basel). 2023 Apr 3;12(7):1537. doi: 10.3390/plants12071537 (PMC10097089; doi:10.3390/plants12071537)
Supplement: Supplementary file 1 [file plants-12-01537-s001.zip › plants-2294420-supplementary.pdf]

Supplementary Materials

Phytochemical analysis and genotoxicological evaluation of prickly pear peel extracts

Margarita Dormousoglou [1], Ioanna Efthimiou [1], Maria Antonopoulou [1,*], Stefanos Dailianis [2], Giulia Herbst [3], Dimitris Vlastos [2,**]

[1] Department of Sustainable Agriculture (Former Department of Environmental Engineering), University of Patras, Seferi 2, GR-30100, Agrinio, Greece

[2] Department of Biology, University of Patras, GR-26500, Patras, Greece

[3] Department of Chemical Engineering, Federal University of Paraná, CEP 81531-990, Curitiba, PR, Brazil

*Corresponding author

**Corresponding author

Email addresses: mantonop@upatras.gr (M.A.), dvlastos@upatras.gr (D.V.)

SM. Results

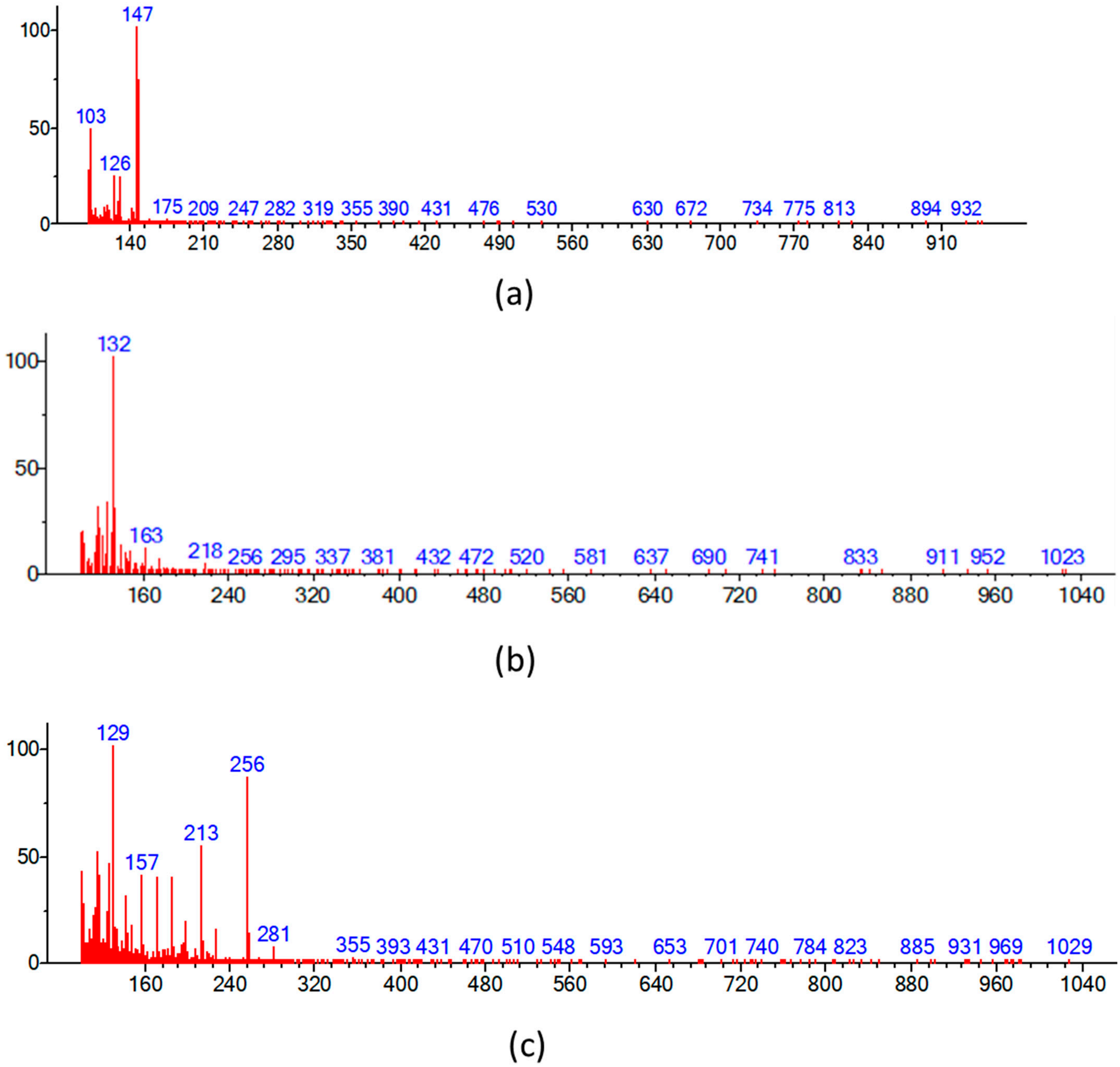

Figure S1. Mass spectra obtained by GC-MS analysis of (a) Trans-Cinnamic acid, (b) Debrisoquine, (c) n-Hexadecanoic acid (Palmitic acid)

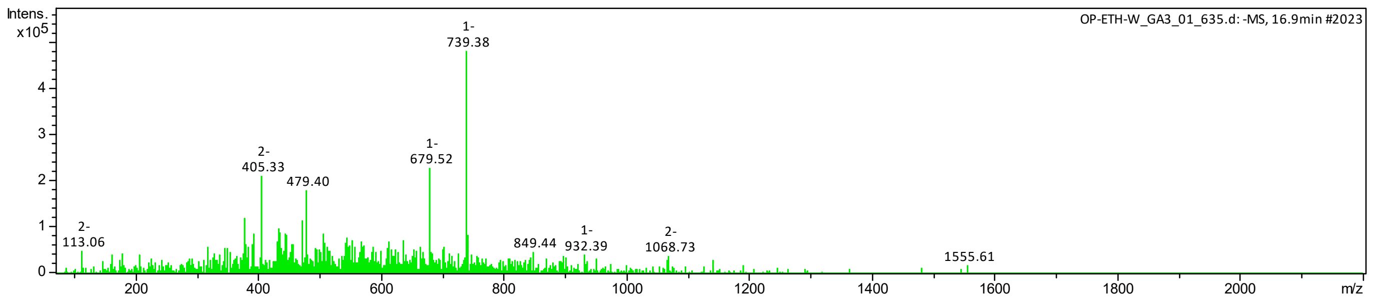

Intens.
x10^5
OP-ETH-W_GA3_01_635.d: -MS, 16.9min #2023
1-
739.38
2-
405.33
479.40
1-
679.52
2-
113.06
849.44
1-
932.39
2-
1068.73
1555.61
m/z

(a)

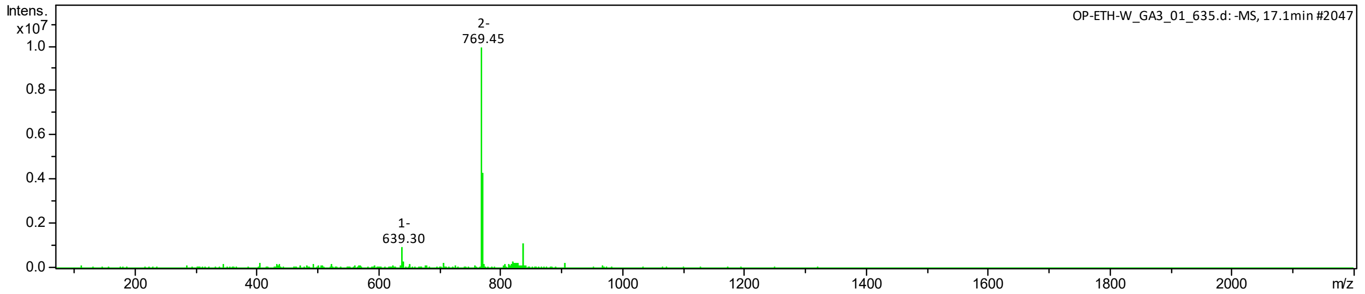

Intens.
x10^7
OP-ETH-W_GA3_01_635.d: -MS, 17.1min #2047
2-
769.45
1-
639.30
m/z

(b)

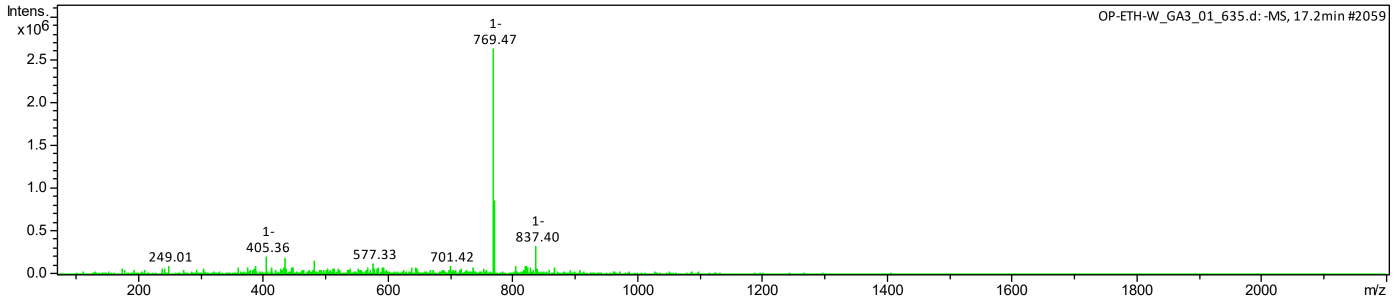

Intens.
x10^6
OP-ETH-W_GA3_01_635.d: -MS, 17.2min #2059
1-
769.47
249.01
1-
405.36
577.33
701.42
1-
837.40
m/z

(c)

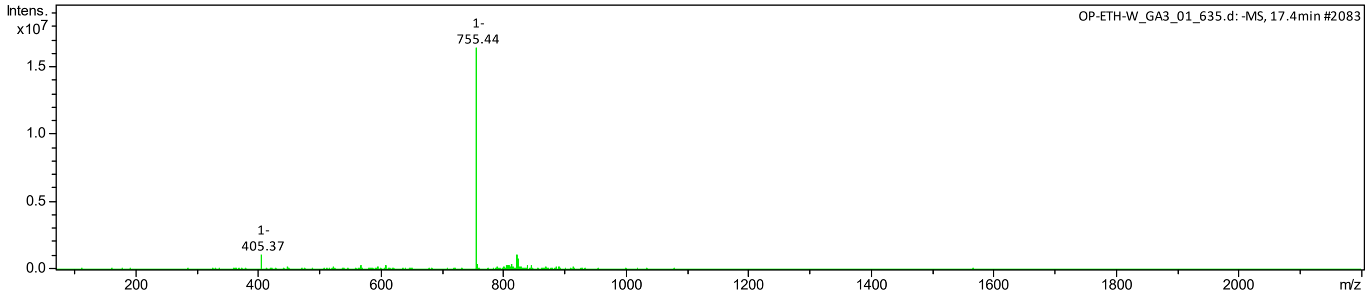

Intens.
x10^7
OP-ETH-W_GA3_01_635.d: -MS, 17.4min #2083
1-
755.44
1-
405.37
m/z

(d)

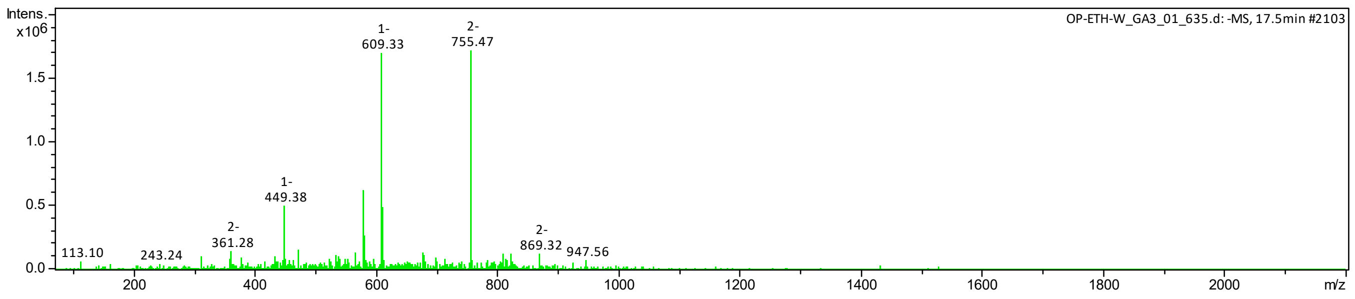

(e)

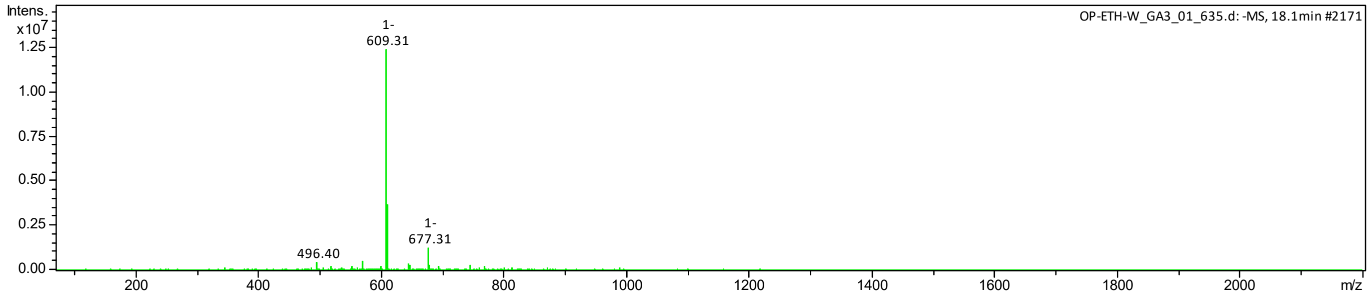

(f)

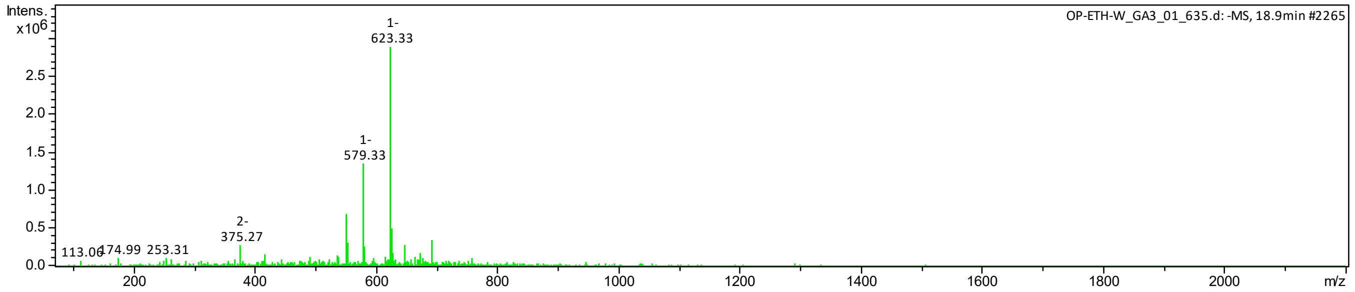

(g)

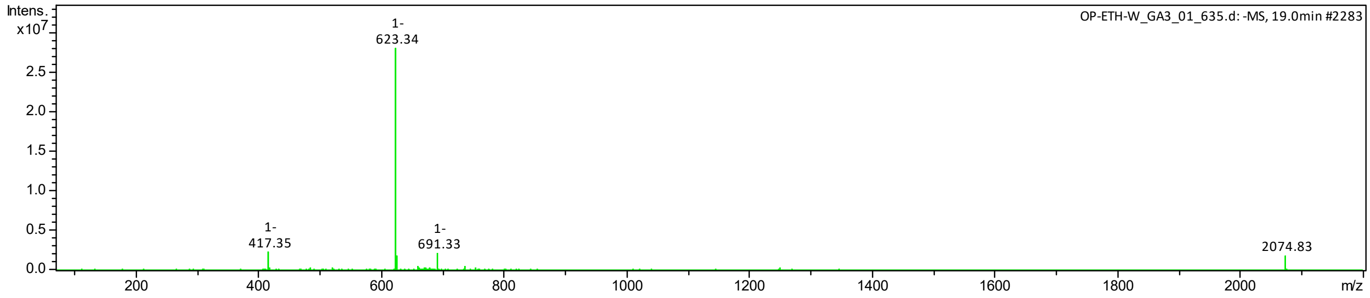

(h)

Figure S2. Mass spectra obtained by UHPLC-MS in negative ionization mode of (a) tri-glycosylated kaempferol (b) tri-glycosylated methyl-quercetin derivative I (c) tri-glycosylated methyl-quercetin derivative II (d)tri-glycosylated quercetin I (e)tri-glycosylated quercetin II (f) di-glycosylated quercetin (Rutin), (g) di-glycosylated methyl-quercetin I, (h) di-glycosylated methyl-quercetin I

SM. Materials and Methods

SM. 4.2 Soxhlet extraction

Specifically, 5 g of dried peel and 150 mL of solvent were added, and the system was heated until boiling. Methanol, ethanol and ethanol/water (ratio 4:1) were used as solvents and the extraction lasted for 6 hrs and was performed in triplicate. Subsequently, the final extracts were concentrated in a rotary vacuum evaporator (Ika HB 10, Germany) and dried using an air circulation oven (IKA, Nova Itica, model 400-2). The extraction yields for each extract (P1: extract obtained with methanol, P2: extract obtained with ethanol, and P3: extract obtained with ethanol/water) were calculated from the following equation:

$$Yield\ (\%) = \frac{mass\ of\ dried\ extract\ (g)}{mass\ of\ sample\ (g)} x\ 100$$

SM. 4.3.1 Total phenolic content (TPC)

Aliquots of P1-P3 extracts were combined with methanol:H_2O to reach 0.5 mL in volume and were mixed with 2.5 mL of Folin-Ciocalteu reagent (diluted 1:10 in distilled water). Then, the mixture was incubated at room temperature in dark for 3 min. Subsequently, 2 mL of 7.5 % Na_2CO_3 were added and the mixtures were incubated in the dark for 2 h. The absorbance of the resulting blue color was measured at 760 nm using a UV-Vis spectrophotometer (Global Analyzer). Calibration curves with concentrations of gallic acid as standard were used for quantification. The gallic acid was used as a standard reference, all determinations were performed in triplicate and results were expressed as milligrams of gallic acid equivalents (GAE) per 1 g of extract (mg GAE/ g^{-1}) ± standard deviation (Singleton et al., 1999).

SM. 4.3.2 Total flavonoid content (TFC)

Specifically, 1.8 mL of distilled water and 0.12 mL of $NaNO_2$ (5% w/v) were added to 0.2 mL of each extract (P1-P3) and mixed thoroughly for 5 min. Thereafter, 0.12 mL of $AlCl_3$ (10% wt/vol) were added, followed by 0.8 mL of NaOH (1 mol L^{-1}) and 0.96 mL of dH_2O. After 5 min, the absorbance was measured spectrophotometrically at 510 nm. Catechin was used for the calibration curve and the results were expressed as mg of catechin equivalent (CE) per 1g of sample (mg CE g^{-1}) (Zhishen et al., 1999).

SM. 4.3.3 Antioxidant activity (AA)

The free-radical-scavenging activity was determined by ABTS (2,2-azino-bis-(3-ethylbenzotiazoline-6-sulfonic acid) and the method was performed according to the procedure described by Re et al. (1999). The radical scavenging activity by DPPH• assay was performed based on the method described by Brand-Williams et al. (1995). The ferric-reducing antioxidant power (FRAP) assay was conducted according to Benzie and Strain (1996). The results were obtained in triplicate. Quantifications were conducted using a Trolox analytical curve and all the results of AA were expressed as μmol of Trolox equivalents per 1 g of sample (μmol TE g^{-1}).

SM. 4.4.1 GC-MS analysis

The following temperature program was implemented: 50 °C, 2 min, followed by ramp of 10° C/min, up to a final temperature of 280 °C, 40 min. The total run was 65 min. The other parameters were as follows: injector temperature: 300 °C, transfer line temperature: 280 °C, MS source temperature: 230 °C, MS quadrupole temperature: 150 °C. NIST MS Search 2.0 was used for compound identification.

SM. 4.4.2 UHPLC/MS analysis

The analysis was performed on an Acclaim RSLC 120 C18, 2,2 μm 120Å (2,1 x 150 mm) column at 30°C and a flow rate of 0.3 mL/min. The mobile phase consisted of water with 0.5% formic acid (A) and acetonitrile with 0.5% formic acid (B). The following gradient program was used: 10% B (0 min), 5% B (10 min), 30% B (20 min), 50% B (30 min), 50% B (32 min), 10% B (38 min) and 10% B (45 min).

SM. 4.5.1 CBMN assay application

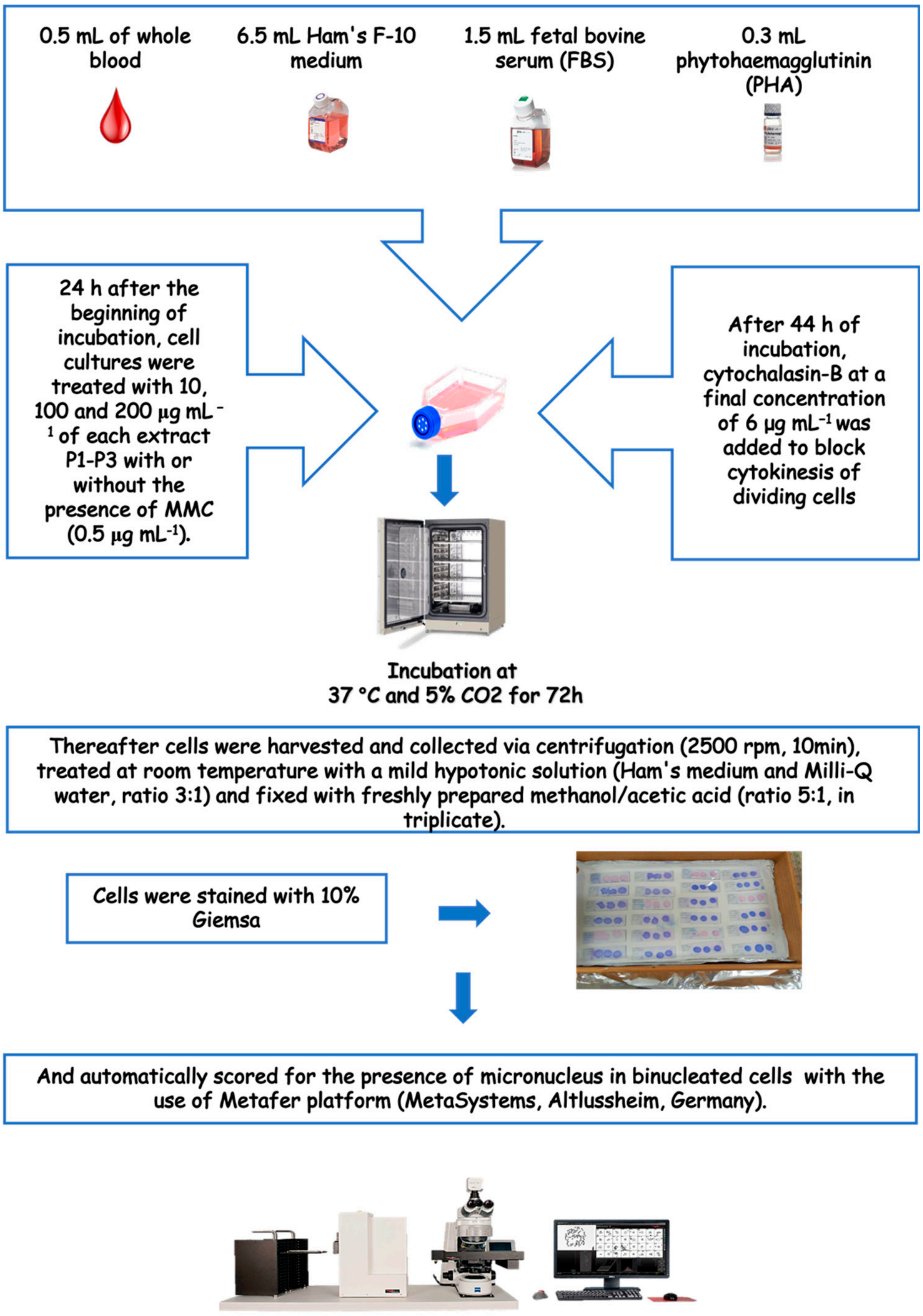

Figure S3. Schematic representation of CBMN assay.

SM. References

Singleton, V.L.; Orthofer, R.; Lamuela-Raventos, R.M. Analysis of total phenols and other oxidation substrates and antioxidants by means of Folin-Ciocalteu reagent. *Methods Enzymol.* **1999**, *299*, 152-178. https://doi.org/10.1016/S0076-6879(99)99017-1

Zhishen, J.; Mengcheng, T.; Jianming, W. The determination of flavonoid contents in mulberry and their scavenging effects on superoxide radicals. *Food Chem.* **1999**, *64*, 555-559. https://doi.org/10.1016/S0308-8146(98)00102-2

Re, R.; Pellegrini, N.; Proteggente, A.; Pannala, A.; Yang, M.; Rice-Evans, C. Antioxidant activity applying an improved ABTS radical cation decolorization assay. *Free Radic. Biol. Med*. **1999**, *26*, 1231-1237. https://doi.org/10.1016/S0891-5849(98)00315-3

Brand-Williams, W.; Cuvelier, M.E.; Berset, C. Use of a free radical method to evaluate antioxidant activity. *LWT- Food Sci. Technol*. **1995**, *28*, 25-30. https://doi.org/10.1016/S0023-6438(95)80008-5

Benzie, I.F.F.; Strain, J.J. The ferric reducing ability of plasma as a measure of "antioxidant power": the FRAP assay. *Anal. Biochem.* **1996**, *239*, 70-76. https://doi.org/10.1006/abio.1996.0292
